# Molecular detection of *Theileria sergentii/orientalis/buffeli* and *Ehrlichia canis* from aborted ovine and caprine products in Sardinia, Italy

**DOI:** 10.1002/vms3.510

**Published:** 2021-05-06

**Authors:** Valentina Chisu, Federica Loi, Lorena Mura, Antonio Tanda, Giovanna Chessa, Giovanna Masala

**Affiliations:** ^1^ Istituto Zooprofilattico Sperimentale della Sardegna ‘G. Pegreffi’ Sassari Italy; ^2^ Osservatorio Epidemiologico Veterinario Regionale, Istituto Zooprofilattico Sperimentale della Sardegna Cagliari Italy

**Keywords:** abortion, *Chlamydia abortus*, *Coxiella burnetii*, *Ehrlichia canis*, infectious agents, *Theileria complex*, *Toxoplasma gondii*

## Abstract

The economic impact and losses caused by abortion of small ruminants represent an important threat to livestock industry worldwide. Infectious agents are the most commonly causes of small ruminant abortion and many of which pose a serious threat to human health. The management of abortion outbreaks is essential to understand the transmission, prevention and control of the zoonotic diseases. This study aimed to increase the knowledge about the common known zoonotic pathogens causing abortion (*Coxiella burnetii*, *Chlamydia abortus* and *Toxoplasma gondii*) circulating in Sardinia. In addition, the occurrence of other infectious agents that, until now, had never been identified in abortion samples and which might be cocirculating during the abortion outbreaks were also considered. In this study, 125 abortion samples collected from 91 small ruminant farms were screened for the presence of *Babesia*/*Theileria* spp., *Ehrlichia canis*, *Anaplasma* spp., *Chlamydia* spp., *C*. *burnetii* and *T*. *gondii* by PCR analyses and sequencing. This is the first evidence on the presence of *Theileria sergenti*/*orientalis*/*buffeli* group and *Eh*. *canis* in 22 (22/125; 18%) and 26 (26/125; 21%) abortion products from small ruminants, respectively. *Chlamydia abortus*, *C*. *burnetii* and *T*. *gondii* were also detected in brain, liver, spleen and placentae at 46% (58/125), 34% (42/125) and 2% (2/125), respectively. This study highlights that pathogens with epizootic and zoonotic potential are circulating in the island and could be involved directly or in association with other pathogens as possible cause of ruminant abortion. Further studies are needed to fully assess the impact of *Theileria sergenti/orientalis*/*buffeli* group and *Eh*. *canis* on ruminant abortion and their real zoonotic risk in the island.

## Graphical abstract

DNAs of Theileria sergenti/orientalis/buffelli and Ehrlichia canis were detected for the first time in ruminant abortion products. The 51% of analyzed samples resulted positive for at least one of the pathogens searched and the 40% of samples resulted co‐infected. Positive and statistically significant index of co‐infection (Ic) were detected in double infection by E. canis/C. burnetii (5.581; *p* = 0.007), Theileria complex/C. abortus (9.713; *p* = 0.005) and Theileria complex/C. burnetii (9.391; *p* = 0.012) from sheep samples. This study indicates that pathogens with epizootic and zoonotic potential are circulating in the island and could be involved directly or in association with other pathogens as possible cause of ruminant abortion.

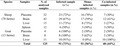

## 1 INTRODUCTION

Abortion in small ruminants is caused by a wide range of infectious agents, and it represents an important cause of economic losses of livestock industries over the world (Benkirane et al., 2015; Kardjadj et al., 2016).

The economic losses are evaluated from production losses, treatment and control costs associated with abortion events. Monitoring the causes of abortion is important for both protecting the healthiness of livestock production and for avoiding the onset of diseases that can be transmissible to humans, especially when the causative agents are zoonotic (van Engelen et al., 2014; Gebretensay et al., 2019). The infectious causes of abortion are not always easily diagnosed, and in most cases, the etiologic agents causing abortion remain unknown (Anderson, 2007). The improper or inadequate specimen collection, handling process and some factors linked to the autolysis process of the organs or environmental contamination, could also prejudge the final diagnosis of abortion in small ruminants. In Sardinia, sheep and goat farms currently account for about one third of value in national ruminant farms, registering more than 3 million of sheep and 0.3 million of goats (National Italian Database 2020 (BDN) June 2020, established by Ministry of Health at the National Surveillance Centre of the IZS in the Abruzzo and Molise Region). Annual losses due to ruminant abortion are estimated around 10 million euros per year. In a previous study, *Toxoplasma gondii*, *Salmonella* enterica serotype *Abortusovis*, *Chlamydia abortus* and *Coxiella burnetii* represented the major protozoan and bacterial infectious agents causing abortion in the island (Masala et al., 2007). In the last decade, some studies conducted in Sardinia highlighted the high diffusion of tick‐borne zoonotic pathogens in arthropods collected from wild and domestic hosts, including sheep and goats (Chisu et al., 2020; Chisu, Foxi, Mannu, et al., 2018, Chisu, et al., 2018). Many tick‐borne diseases cause considerable economic damage to livestock farms as the infection in ruminants can be accompanied by abortion and reducing livestock production (Hurtado & Giraldo‐Ríos, 2018; Kivaria, 2006). In Sardinia, the climatic conditions exposed sheep to the risks of tick infestations throughout whole year (Satta et al., 2011). Since some of the pathogens vectored by ticks have never been investigated in abortion products, we performed a molecular survey with the following aims: (1) to identify infectious causes of abortion by testing all samples with the routinary panel of already known microorganisms‐caused ovine and caprine abortion in Sardinia, (2) to include in this panel the molecular identity of other infectious agents which might co‐circulate during the abortion outbreak and (3) to detect possible co‐infections in the same aborted samples.

## 2 MATERIALS AND METHODS

### 2.1 Sample collection and molecular detection of pathogens

During 2018, 43 foetuses from 78 sheep flocks and 11 from 13 goat herds were collected by veterinary practitioners and then submitted to the molecular laboratory of the Istituto Zooprofilattico Sperimentale della Sardegna to be tested for detection of infectious agents known as potential causes of abortion and circulating in Mediterranean area (Chisu et al., 2020). Thirty‐two and four placentae from sheep and goats, respectively, were also collected in this study.

Brain, liver and spleen were collected from each foetus wherever possible. However, the condition of some of the foetuses was such that not all tissues could be collected. In fact, dogs or wild animals can frequently eat the placenta and occasionally consume dead foetuses or parts of them. Moreover, factors such as the inadequate transport conditions, autolysis and other causes may adversely affect the collection. Placental samples were homogenated by using the cotyledon and intercotyledon regions (Masala et al., 2007). Foetal organs and placental samples were washed with phosphate buffered saline (PBS) containing 1,000 U/ml of penicillin and 1,000 U/ml of streptomycin sulfate and then digested with 2% trypsin for 3 hr at 37°C. The brain was digested by using trypsin at 0.6%. Digested tissues were then filtered through sterile gauze and centrifuged at 3,000 × *g* for 10 min. After three washes, pellets were resuspended in Foetal Bovine Serum (FBS) containing 10% dimethylsulphoxide, aliquoted, and stored at −20°C until use (Masala et al., 2007). As part of the routinary diagnostic methods available for the abortion diagnosis of the Istituto Zooprofilattico Sperimentale della Sardegna, all collected samples were tested for the presence of bacterial (*Anaplasma* spp., *Chlamydia* spp., *Coxiella burnetii*, *Ehrlichia canis*,) and protozoal (*Toxoplasma gondii*, and *Babesia*/*Theileria* spp.) agents by PCR analyses as previously described (Chisu et al., 2020; Masala et al., 2007).

### 2.2 Purification and sequencing of positive samples

To identify detected pathogens, positive PCR products were purified and sequencing was performed as described in previous studies (Chisu et al., 2020; Masala et al., 2007). All sequences obtained were assembled and edited with Chromas pro software version 2.6. and were then analysed by BLAST (www.ncbi.nlm.nih.gov/blast/Blast.cgi) sequencing analysis in the GenBank database.

### 2.3 Statistical analysis

Based on positive/negative samples, contingency 2 × 2 tables were performed for each pathogen and Chi‐square test was used to assess significance. To quantify the degree of departure of the number of mixed infections from independence, index of co‐infection (Ic) was used (Ginsberg, 2008). All statistical analyses were performed using Stata 13 [Stata‐Corp, Stata statistical software: release 13, StataCorp LP, College Station, TX, USA (2013)] and R‐opensource (Version 3.3.2, R‐foundation for Statistical Computing, Vienna, Austria), setting a statistically significant value of 0.05.

## 3 RESULTS

A total of 125 abortion products were collected from 91 different small ruminant farms located in several sites from Sardinia island. Overall, 36 placentae (32 from sheep and 4 from goats), 51 brains (43 from sheep and 8 from goats), 26 liver samples (15 from sheep and 11 from goats) and 12 spleen samples from sheep were collected as illustrated in Table 1. Over 125 total samples, 51 (56%) were infected with at least one of the following pathogens: *Babesia/Theileria* spp., *T*. *gondii*, *E*. *canis*, *C*. *burnetii*, and *Chlamydia* spp., whilst 40/125 (44%) samples were co‐infected with two or more pathogens as illustrate in Table  1.

**TABLE 1:** PCR screening results from sheep and goats abortion samples collected

Species	Samples	No. of analysed samples	Infected sample (*n*.%)	Mono‐infected samples (*n*.%)	Co‐infected samples (*n*.%)
Sheep (78 farms)	Placenta	32	23 (72%)	16 (70%)	7 (30%)
	Brain	43	29 (67%)	17 (59%)	12 (41%)
	Liver	15	11 (73%)	8 (73%)	3 (27%)
	Spleen	12	6 (50%)	3 (50%)	3 (50%)
Goat (13 farms)	Placenta	4	4 (100%)	2 (50%)	2 (50%)
	Brain	8	8 (100%)	5 (62%)	3 (38%)
	Liver	11	10 (91%)	0 (0%)	10 (100%)
	Total	125	91 (73%)	51 (56%)	40 (44%)

Amongst protozoal species, *Babesia/Theileria* spp. were the most pathogen detected by conventional PCR, followed by *T*. *gondii* as shown in Table 2. Specifically, *Babesia*/T*heileria* spp. were identified in 22 samples as shown in Table 2. Abortion products from goats resulted most frequently positive for piroplasmids species (10%; 12/125), followed by sheep abortion samples (8%; 10/125). Sequencing of the 18S rRNA performed on positive amplicons showed that all sequences detected from both sheep and goats abortion samples were 100% identical to that of *Theileria sergenti/orientalis*/*buffeli* strains, isolated worldwide. *Theileria* group DNA was mainly found from sheep brain (5/43; 12%) and liver (3/15; 20%) samples and from goat liver tissues (9/11; 82%) as shown in Table 2.

**TABLE 2:** Summary of infectious agents detected by PCR from abortion samples related to the total of them

Microorganism	Sheep				Goat		
	Placenta (*n* = 32)	Brain (*n* = 43)	Liver (*n* = 15)	Spleen (*n* = 12)	Placenta (*n* = 4)	Brain (*n* = 8)	Liver (*n* = 11)
*Babesia/Theileria* spp.	—	5	3	2	2	1	9
*Toxoplasma gondii*	2	—	—	—	—	—	—
*Chlamydia* spp.	14	15	7	4	4	4	10
*Coxiella burnetii*	18	10	3	2	2	2	5
*Ehrlichia canis*	3	12	2	1	—	5	3

The presence of DNA from *T*. *gondii* was detected in two placentae (2/125; 1.6%) from sheep by nested PCR assays targeting 227 bp of the multicopy 18S‐5.8S rRNA internal transcribed spacer (ITS1) region.

Amongst bacterial species, PCR positivity was higher for *Chlamydia* species that were the most frequently isolated (58/125; 46%), followed by *C*. *burnetii* detected in 42/125 (34%) samples and *E*. *canis* detected in 26/125 (21%) ruminant abortion samples examined (Table 2).

Chlamydial DNA was mostly detected in brain samples (15/43; 35%) and placentae (14/32; 44%) from sheep abortion products, and in placenta (4/4; 100%), brain (4/8; 50%) and liver samples (10/11; 91%) from goat abortion products. Conventional PCR and sequencing targeting the partial 16S rRNA gene of *Chlamydia* spp., returned clear chromatogram signals in 29/58 amplicons. The remaining 29 samples resulted unreadable probably suggesting multiple *Chlamydiales* agents are present in aborted samples. Following BLAST analyses used to determine the likely sequence identity, all the sequences detected were found to share 100% identity to the partial 16S rRNA gene sequences from *Chlamydia abortus* strains.

Three placentae (3/32; 9%) from sheep, 17 brain samples (17/51; 33%), 5 liver samples (5/26; 19%) and 1 (1/12; 8%) spleen from sheep were positive for Ehrlichial DNA. Amplified fragments were sequenced and showed 100% identity with the major outer membrane protein P30 fragment gene of *E*. *canis* strains.

C*oxiella burnetii* DNA was detected from 18 sheep placenta samples (18/32; 56%) and 2 (2/4; 50%) from placenta goats, 10 brain samples (10/43; 23%) from sheep and 2 (2/8; 25%) from goats, 3 liver samples (3/15; 20%) derived from sheep and 5 (5/11; 45%) from goats and 2 (2/12; 17%) spleen samples from sheep. The DNA sequence and BLAST analyses revealed 100% sequence identity with the corresponding 257‐bp region of the *C*. *burnetii* superoxide dismutase (SOD) gene. No *Anaplasma* DNA was detected by PCR in any of the tested samples.

Concomitant infections with two or more pathogens were simultaneously detected in placenta, brains, liver and spleen as described in Table 3.

Specifically, seven, three and one combination of co‐infections with two, three and four different pathogens were detected in sheep abortion samples, respectively. Instead, three, two and one combination of co‐infections with two, three and four different pathogens, were detected in goat abortion samples, respectively.

In sheep, the most frequent but not statistically significant co‐infection was those of *C*. *abortus* and *C*. *burnetii*, detected in 13 samples: 7 brains, 1 liver and 5 placentas. In goats the co‐infection of *Theileria* complex and *C*. *abortus* has been detected in 1 brain, 9 livers and 2 placentas. Positive and statistically significant index of co‐infection (Ic) was detected in double infection by *E*. *canis/C*. *burnetii* (5.581; *p* = 0.007) in sheep brain samples, *Theileria* complex/*C*. *abortus* (9.713; *p* = 0.005) in brain, liver and spleen samples and *Theileria* complex/*C*. *burnetii* (9.391; *p* = 0.012) in brain, liver and spleen from sheep. No one statistically significant Ic has been detected in samples from goats (Table 3).

**TABLE 3:** Statistical analysis of double and multiple co‐infection rates

Species	Co‐infection	Placenta	Brain	Liver	Spleen	Ic	*p*
Sheep	*Eh*. *canis*/*C*. *burnetii*	—	8	—	—	5.581	0.007
	*Theileria* complex/*C*. *abortus*/*Eh*. *canis*/*C*. *burnetii*	—	1	—	—	ns	ns
	*Theileria* complex/*C*. *abortus*/*C*. *burnetii*	—	3	1	—	ns	ns
	*Theileria* complex/*C*. *abortus*	—	5	2	1	9.713	0.005
	*Theileria* complex/*C*. *burnetii*	—	4	2	1	9.391	0.012
	*C*. *abortus*/*Eh*. *canis*/*C*. *burnetii*	—	2	—	—	ns	ns
	*C*. *abortus*/*C*. *burnetii*	5	7	1	—	−1.171	ns
	*C*. *abortus*/*Eh*. *canis*	2	3	—	1	−2.031	ns
	*C*. *abortus*/*T*. *gondii*/*C*. *burnetii*	1	—	—	—	ns	ns
	*T*. *gondii*/*C*. *burnetii*	2	—	—	—	3.753	ns
	*Theileria complex/ Eh*. *canis*	—	1	—	—	−2.837	ns
Goat	*Eh*. *canis*/*C*. *burnetii*	—	1	3	—	6.689	ns
	*Theileria* complex/*C*. *abortus*/*Eh*. *canis*/*C*. *burnetii*	—	—	3	—	ns	ns
	*Theileria* complex/*C*. *abortus*/*C*. *burnetii*	2	—	2	—	ns	ns
	*Theileria* complex/*C*. *abortus*	2	1	9	—	0.986	ns
	*Theileria* complex/*C*. *abortus*/*Eh*. *canis*	—	1	—	—	ns	ns
	*C*. *abortus*/*Eh*. *canis*	—	1	—	—	−6.004	ns

Note

*p*, probability from chi‐square test, Ic, index of co‐infections.

## 4 DISCUSSION AND CONCLUSION

In Sardinia, ovine and caprine livestock industries represent the top in the agricultural sector. Although abortion in small ruminants has considerable economic impact on the productivity of herds in Sardinia, the implementation of analyses to ascertain the putative pathogens‐causing abortion needs to be improved. The continuous and reliable recording of the distribution of ruminant abortions in the island is necessary as well as the identification of the source and route of infection that in some cases remains largely unknown. It has been estimated that only in 30% of cases it is possible to identify the etiological agent causing abortion. Recently, several publications provided evidence that bacterial and protozoal agents are circulating in arthropods, especially in ticks (Chisu et al., 2020). The presence of *T*. *sergenti/orientalis*/*buffeli* group in aborted samples from Sardinia has been demonstrated for the first time in this study. This complex causes economic losses mainly in affected cattle herds where it could determine abortions, reduction of milk productions and severe morbidity and mortality rates (Hornok et al., 2014; Kumsa et al., 2014; Sivakumar et al., 2014; Watts et al., 2016).

To date, *T*. *sergenti/orientalis*/*buffeli* group has been also recorded in sheep, ticks and other blood feeding insects, worldwide (Chisu et al., 2020). Since *T*. *buffeli*/*sergenti*/*orientalis* group is transmitted principally by Ixodid ticks of the genus *Haemaphysalis*, *Amblyomma* and *Rhipicephalus* (Hornok et al., 2014; Kumsa et al., 2014; Toma et al., 2017), the high frequency of this group in aborted samples could be explained by the abundance and wide distribution of their competent vectors in the Island that are constantly reported in Sardinia (Chisu et al., 2020; Satta et al., 2011). In Sardinia, *T*. *sergenti/orientalis*/*buffeli* group DNA has been detected in several tick species including *Hae*. *punctata*, *Rh*. *annulatus* and *D*. *marginatus* collected from cattle and goats (Chisu et al., 2019). A recent study showed that *T*. *sergenti/orientalis*/*buffeli* group was also detected in asymptomatic and symptomatic calves and goats from Sardinia (Zobba et al., 2020). In addition, the transplacental transmission of *T*. *orientalis* in cattle has been proposed (Baek et al., 2003; Swilks et al., 2017), and evidence of the complex in colostrum samples suggests also a transmammary transmission (Hammer et al., 2016). Further research is required to determine if *T*. *sergenti/orientalis*/*buffeli* group could be considered the causal agent of ovine/caprine abortion or a collateral cause in co‐presence with other microorganisms.

As far as we know, this study reported the first molecular detection of *E*. *canis* from placenta and foetal organs from both sheep and goats flocks from Sardinia. Although *Eh*. *canis* is known as the pathogen that commonly infects dog worldwide, infections have also been described in sika deer (Li et al., 2016), goats (Zhang et al., 2017), humans (Maeda et al., 1987; Perez et al., 2006), cats (Braga et al., 2014) and arthropods (Sainz et al., 2015) and there is thus growing evidence that *Eh*. *canis* has a wider host range than previously thought.

*Ehrlichia canis* is mainly vectored by *R*. *sanguineus* s.l., which is largely present in the Mediterranean basin where *E*. *canis* is endemic (Sainz et al., 2015). In previous studies, the presence of *Eh*. *canis* was highlighted in *Rh*. *sanguineus* s.l., *Hy*. *marginatum*, *Rh*. *bursa* and *Hy*. *marginatum* ticks collected from sheep and goats (Chisu, Foxi, Mannu, et al., 2018). Although *Eh*. *canis* pathogenicity in domestic ruminants is still unknown, detection of this microorganism in abortion products warrants further investigations.

The results of this study indicate that *Ch*. *abortus* DNA was the most frequently detected bacterial pathogen in abortion products. The intracellular bacteria *Ch*. *abortus*, the causative agent of enzootic abortion of ewes (EAE), affects the placenta of sheep and goats leading to abortion in late gestation (Essig & Longbottom, 2015). This pathogen is mainly of veterinary importance being involved in abortion of ruminants as well as other domestic and wild mammals, reptiles and amphibians (Opota et al., 2015). *C*. *abortus* represents also a zoonotic risk to humans since it can colonise the human placenta and lead to foetal death and miscarriage (Essig & Longbottom, 2015; Opota et al., 2015). The last report of the pathogen in the island dates back to 2007 when it was detected in 12.5% of placentae from caprine aborted samples and from 2.4% of foetuses and 14.4% of placentae of ovine aborted samples (Masala et al., 2007). Recent studies have described the presence of *C*. *psittaci*, *C*. *abortus* and *Parachlamydia acanthamoebae*, known for their potential as human and veterinary bacterial pathogens, in *Rhipicephalus*, *Haemaphysalis* and *Dermacentor* tick genera from Sardinia (Chisu, et al., 2018). Ruminants are hosts to a wide range of tick species that vectors members of the order *Chlamydiales*, including *C*. *abortus*. Since *C*. *abortus* strains detected in aborted samples are the same detected in ticks, their involvement as routes of transmission of *C*. *abortus* is yet to be fully defined. However, considering the significant economic losses in farms due to this pathogen, the early and accurate diagnosis of *C*. *abortus* is necessary, so that appropriate control measures can be adopted to limit or prevent the spread of infection.

*Coxiella burnetii* is another important infectious agent of Sardinian livestock, causing significant economic losses to the livestock industry (Masala et al., 2004). Domestic mammals, mainly goats, sheep, and cattle, are the most important reservoir of infection of this bacterium. Transmission from animal reservoirs to humans occurs primarily through the inhalation of contaminated aerosols (Schneeberger et al., 2014). In this study, the presence of *C*. *burnetii* was also confirmed in abortion products. *C*. *burnetii* was mainly detected in placentae (18/32; 56%) and in brain samples (10/43; 23%) from sheep and in liver organs from goats (5/11; 45%) with a prevalence rate of infection that was higher than any previously reported in the island (Masala et al., 2007). Moreover, recent studies identified *C*. *burnetii* in *R*. *sanguineus* s.l., *R*. *annulatus* and *H*. *marginatum* ticks from Sardinia (Chisu, Foxi, Mannu, et al., 2018; Chisu et al., 2020) confirming that it is likely that arthropods could act as vectors of the pathogenic *C*. *burnetii* in Sardinian ruminants.

Toxoplasmosis, a disease caused by *Toxoplasma gondii*, is an important zoonosis and a major cause of abortion in sheep (Zedda et al., 2010). In this study, *T*. *gondii* has been detected in very low numbers and only two cases have been reported in ovine placental samples (2/32; 6%). In 2007, a survey of major infectious causes of abortion in small ruminants in Sardinia highlighted that *T*. *gondii* was the most frequently detected pathogen (18.1% of foetuses and 13.1% of placentae from sheep aborted samples; 13% of foetuses and 25% of placentae from goat aborted samples), indicating its important role in ovine and caprine abortions (Masala et al., 2007). These results suggest that the adequate management of the Sardinian farms, including regular rodent control actions and the isolation of the abortion ewes, has allowed to the control of abortion outbreaks caused by *T*. *gondii*. Considering the strong relevance of toxoplasmosis for human health, further studies are needed in order to continue to monitoring the pathogen.

Pathogens causing abortions can coexist and cause concurrent infections with more than one pathogen and can increase the risk of atypical forms of clinical disease. These results highlight that two or more pathogens were simultaneously present in placenta, brains, liver and spleen indicating that ovine and caprine farms are exposed to multiple pathogens. Since one or more pathogens can coexist in the same tick, we can suppose that the arthropods could vector and transmit more pathogens simultaneously to the host (Chisu et al., 2020). Co‐infecting pathogens might alter the efficiency of transmission, cause cooperative or competitive pathogen interactions and alter disease severity amongst hosts (Swanson et al., 2006). So the direct responsibility of the single pathogens in abortion could not be definitively established without the confirmation of the presence of the lesions caused by the different infectious agents by macroscopic, microscopic and histological examination of the abortion products. These information will be of interest to laboratory diagnosticians that should consider the possibility that other abortifaciens agents are circulating in the island and could be the cause of ovine and caprine abortion.

## CONFLICT OF INTEREST

The authors declare that they have no conflict of interests.

## AUTHOR CONTRIBUTION

**Valentina Chisu:** Conceptualization; Data curation; Investigation; Methodology; Supervision; Writing‐original draft; Writing‐review & editing. **Federica Loi:** Data curation; Formal analysis; Methodology; Software. **Lorena Mura:** Data curation; Formal analysis; Investigation; Methodology. **Antonio Tanda:** Data curation; Formal analysis; Investigation; Methodology. **Giovanna Chessa:** Data curation; Formal analysis; Investigation; Methodology. **Giovanna Masala:** Project administration; Supervision; Writing‐review & editing.

## ETHICS APPROVAL

The authors declare that all applicable international, national and/or institutional guidelines for the care and use of animal were followed.

### PEER REVIEW

The peer review history for this article is available at https://publons.com/publon/10.1002/vms3.510.

## Data Availability

Not applicable.
